# Dietary Intake Reporting Accuracy of the Bridge2U Mobile Application Food Log Compared to Control Meal and Dietary Recall Methods

**DOI:** 10.3390/nu11010199

**Published:** 2019-01-19

**Authors:** Jennifer L. Lemacks, Kristen Adams, Ashley Lovetere

**Affiliations:** The University of Southern Mississippi, 118 College Drive #5142, Hattiesburg, MS 39406-0001, USA; anna.k.lee@usm.edu (K.A.); ashley.lovetere@usm.edu (A.L.)

**Keywords:** diet, assessment, food log, recall

## Abstract

Mobile technology introduces opportunity for new methods of dietary assessment. The purpose of this study was to compare the reporting accuracy of a mobile food log application and 24 h recall method to a controlled meal among a convenience sample of adults (18 years of age or older). Participants were recruited from a community/university convenience sample. Participants consumed a pre-portioned control meal, completed mobile food log entry (mfood log), and participated in a dietary recall administered by a registered dietitian (24R). Height, weight, and application use survey data were collected. Sign test, Pearson’s correlation, and descriptive analyses were conducted to examine differences in total and macronutrient energy intake and describe survey responses. Bland Altman plots were examined for agreement between energy intake from control and 24R and mfood log. The 14 included in the analyses were 78.6% female, 85.7% overweight/obese, and 64.3% African American. Mean total energy, protein, and fat intakes reported via the mfood log were significantly (*p* < 0.05) lower compared to the control, by 268.31kcals, 20.37 g, and 19.51 g, respectively. Only 24R mean fat intake was significantly (*p* < 0.01) lower than the control, by 6.43 g. Significant associations (r = 0.57–0.60, *p* < 0.05) were observed between control and mfood log mean energy, carbohydrate, and protein intakes, as well as between control and 24R mean energy (r = 0.64, *p* = 0.01) and carbohydrate (r = 0.81, *p* < 0.001) intakes. Bland Altman plots showed wide limits of agreement, which were not statistically significant but may have practical limitations for individual dietary assessment. Responses indicated the ease of and likelihood of daily mfood log use. This study demonstrates that the Bridge2U mfood log is valid for the assessment of group level data, but data may vary too widely for individual assessment. Further investigation is warranted for nutrition intervention research.

## 1. Introduction

Dietary assessment is a critical component used to identify relationships between nutrients and chronic disease, such as obesity, cancer, cardiovascular disease, and diabetes. Self-report dietary assessment instruments are widely used in research to estimate energy, macronutrient, and micronutrient intakes, and to relate nutrient intake to various psychosocial and clinical factors to determine disease risk and association [1,2]. Compared to gold standard methods that introduce the least bias, such as the doubly labeled water to determine energy expenditure [3], self-report dietary assessment instruments are much less invasive and costly and are easily conducted in real world settings. The accuracy of dietary assessment methods is particularly important for identifying associations between diet and disease and determining changes in dietary behavior as a result of intervention.

Self-report dietary assessment methods include 24-h recalls (24R), food frequency questionnaires, a food record/diary/log, and food screeners. The 24R method is considered the best self-report dietary assessment instrument due to the least bias reported of self-report instruments [1], especially when recalls are interviewer-administered and conducted using the multi-pass method [4]. However, the method is not without limitations, which are largely due to an individual’s inability to recall what they ate the previous day or accurately estimate portion sizes [5]. Additionally, numerous factors have been linked to dietary intake misreporting from self-reported instruments. For example, it is well-known that overweight or obese individuals commonly underreport energy intake compared to normal weight counterparts from self-administered food frequency questionnaires [6,7,8]. 

Mobile technology assisted dietary assessment has emerged as a method for collecting dietary data and allows for real-time recording of food intake [9]. A review of mobile dietary assessment methods examined for feasibility and validity determined three main assessment methods: self-reported dietary intake entry, food photograph analyses by trained research dietitians, and auto-analyzed food images [10]. While electronic food logs may exhibit similar limitations to pen and paper methods (i.e., participant’s inability to estimate portion sizes or recall foods, resulting in omission of foods), mobile food logs offer vast opportunities, including reduced researcher burdens and costs due to automatic, real-time data entry and allow for the real-time detection of procedural issues/non-compliance and communication with participants to alleviate issues [10]. Despite the potential, there is limited research on the validity of mobile self-report food logs. Thus, the purpose of this study was to examine the reporting accuracy of a mobile dietary recall food log application (a self-reported dietary intake entry method) and 24R method for a controlled meal among a convenience sample of adults (18 years of age or older).

## 2. Materials and Methods

### 2.1. Setting and Technology Development

**Figure 1:**
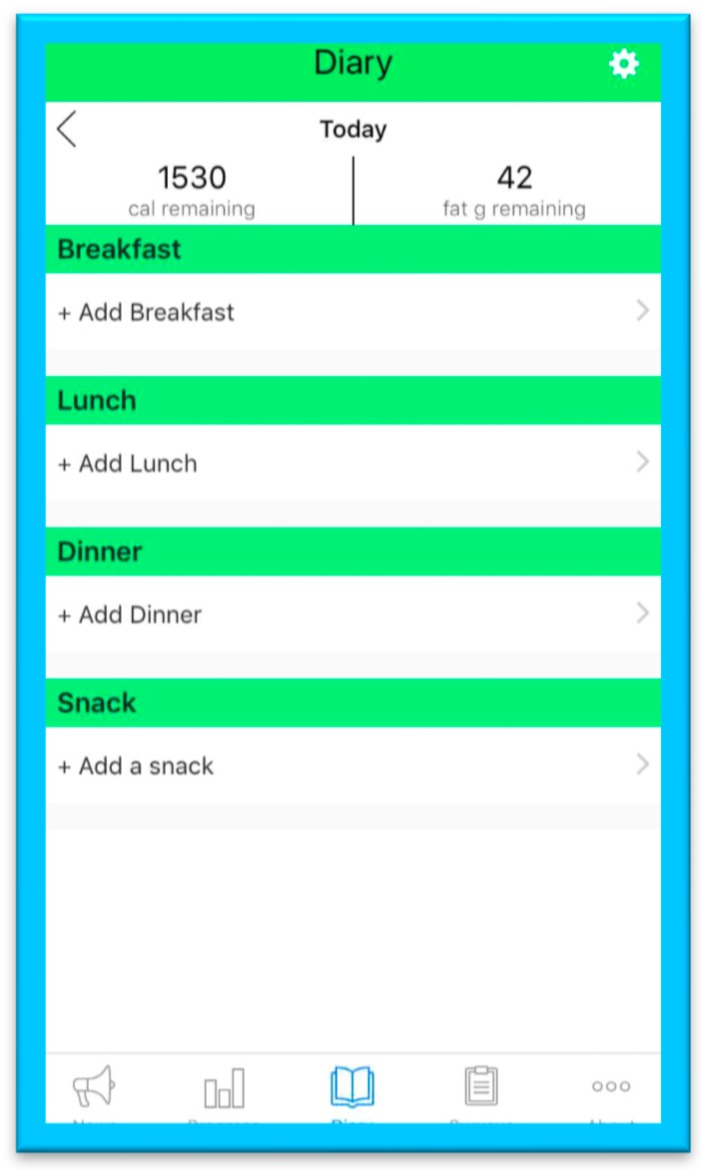
Screen capture of the Bridge2U mobile food log.

The study presented in this manuscript was a secondary study of the Church Bridge Project, which was a weight management intervention delivered in a church-based setting and targeted young to middle aged (18 to 50 years of age) adult African Americans [1]. The intervention included the development of a mobile and web application (Bridge2U) to facilitate survey, anthropometric, and dietary data collection. The platform also allowed interventionists to deliver dietary feedback to participants received via the mobile application and participants to monitor their weight loss progress. A mobile food log (mfood log, depicted in Figure 1) allowed participants to enter dietary data in real time or prospectively and was used for this secondary study. The mfood log utilized the USDA Food Composition Database API [2] to support the search location of foods in the application and includes 7793 standard reference foods and 229,064 branded food products. According to the National Institutes of Health definition, this original study was considered a clinical trial and registered at ClinicalTrials.gov (identifier: NCT02773069).

### 2.2. Recruitment

Two groups were recruited for the study from the Church Bridge Project research sample [1] and university population to result in a community and university convenience sample, respectively. Neither group was currently enrolled in an active dietary intervention. General inclusion criteria were adults 18 years of age or older and either Church Bridge Project participants or university students, faculty, or staff. Participants were excluded from the university sample if they were nutrition or computer science students, faculty, or staff, and individuals that the researchers knew personally. Flyers were utilized to recruit participants in the study, as well as word of mouth through church and university leaders. Interested participants completed an online enrollment form to collect basic contact information, food allergy information, inclusion criteria (age, department), and how they heard about the study. All instruments and protocol were approved by The University of Southern Mississippi Institutional Review Board.

### 2.3. Control Meal Preparation

Researchers and registered dietitians prepared a control meal to include spaghetti and meat sauce; the option for a parmesan cheese topping; steamed broccoli; a roll; a dessert option of a sugar cookie or chocolate pudding; and a beverage choice of Coke, Diet Coke, Sprite, or water. All items were pre-portioned and a nutrient analysis of the pre-portioned items (including the optional cheese) was conducted using the USDA Food Composition Database [11].

### 2.4. Data Collection

Upon arrival to the study site, participants received an oral consent form, a test mobile phone, and random participant identifier to log into the Bridge2U mobile application. After all participants were checked in, a researcher delivered an oral consent presentation and consent was obtained from participants. While there were no hidden video or audio recordings, the researcher provided minimal information regarding the project research questions (i.e., what was being monitored and measured) in an attempt to maintain the integrity of the study and preserve natural behaviors in a controlled setting. Participants were given an overview of the study to include the major study requirements: meal participation, mfood log entry, and 24R administration the following day. Participants were not informed of the purpose for mfood log entry or given specifics as to why the dietitian would be calling them the next day, other than “to conduct a follow up interview”.

### 2.5. Meal Participation, mFood Log Entry and 24R Administration

Prior to meal consumption, participants were instructed to login to the mobile application using a random identifier. Participants were then instructed to enter food items and portions consumed whenever they deemed appropriate during the study meal using the mfood log. The pre-portioned control meal was provided to each participant with the option to request additional pre-portioned servings of any food or beverage offered. Research dietitians recorded consumption as a percentage of each meal component consumed once participants stated they were done consuming their meal and both prior to and after any additional servings were provided. For example, if the participant consumed all of an entrée, the researcher would record 100% of the entrée as consumed. If the participant consumed half of the roll and none of the broccoli, the researcher would record 50% and 0% consumed for those meal components, respectively. 

After completion of the meal and mfood log, participants were reminded that they would be contacted by a registered dietitian the following day to complete a 24R of the study meal. The day after the meal consumption study, a registered dietitian contacted each participant to complete the food recall interview of the study meal consumed following standard, 24R methodology [12]. Data were recorded and analyzed using the USDA Food Composition Database [11], in alignment with the meal analysis and mfood log database used.

### 2.6. Anthropometric and Survey Data

Height and weight data were collected using a portable SECA 217 stadiometer and SECA 869 digital weight scale, respectively, and measured to the nearest tenth. Body mass index (BMI) was calculated as weight in kilograms divided by height in meters, squared. After using the mfood log, participants completed a self-administered survey with Likert response items to determine the ease of use of the application (with a scale of 1 being most difficult and 10 being easiest), likelihood of using the application on a daily basis (with a scale of 1 being least likely to 10 being most likely), and whether they had previously used a dietary intake mobile application (yes or no); responses “yes” to previous use of a dietary intake application were followed by a request to name the application previously used.

### 2.7. Statistical Analysis

Data from the control, mfood log, and 24R methods were analyzed using the USDA Food Composition database [2] to maintain data consistency and determine total energy intake in kilocalories, as well as macronutrient (carbohydrate, fat, and protein) grams consumed. Scatterplots and boxplot analyses were examined for potential outliers. A sign test was conducted to examine mean differences in energy intake between the control meal, 24R, and mfood log data. Pearson’s correlation estimates were examined to note any significant linear associations between total and macronutrient energy intake computed between control, mfood log, and 24R methods and ease and likelihood of use and energy reporting differences between control, mfood log, and 24R; the method was used as an indicator of group level agreement between methods. A Bland Altman plot was used to examine individual level agreement between control and mfood log, which is useful to identify a relationship between differences and magnitude of measurements systematic bias. Descriptive data were reported to describe responses to survey items. All analyses were conducted using IBM SPSS 18.0 (Armonk, NY, USA). Statistical significance was determined based on an alpha level less than or equal to 0.05. 

## 3. Results

Six and 21 participants were reached for the community and university samples, respectively, and invited to participate in the controlled study. Six participants enrolled from the Church Bridge Project and one had to withdraw due to a family emergency. Twenty-one participants enrolled from the university setting; however, three were excluded due to scheduling conflicts, two withdrew for personal reasons, and six did not attend data collection. Therefore, there were five and 10 participants from the church and university settings, respectively. There were 15 participants in total included in the study from both settings. Examination of scatterplots revealed one potential outlier. Boxplot analysis revealed the value was an extreme value (greater than quartile 1 multiplied by 1.5 and added to the interquartile range value). The participant was removed from the sample, resulting in a final 14 participants included in the study.

### 3.1. Participant Characteristics

Participant ages ranged from 19 to 45 years, with a mean of 26.2 years. Participants were 78.6% (*n* = 11) female, 85.7% (*n* = 12) overweight/obese, and 64.3% (*n* = 9) African American; race, gender, and body mass index class counts and percentages are reported in Table 1. 

### 3.2. Inferential Analyses

Reported mean energy intake was lower than the control meal for both the 24R and mfood log. Sign test results showed that there was no significant difference in energy intake reported between 24R and mfood log methods (*p* = 0.18). Mean energy intake reported using the mfood log was statistically significantly (*p* = 0.002) lower than the control meal; 24R was not significantly (*p* = 0.09) lower than the control meal. The mean differences between the mfood log and control method were also significantly lower for protein (*p* < 0.001) and fat (*p* = 0.001) intakes. mFood log mean protein (*p* = 0.01) and fat intakes (*p* = 0.002) were also significantly lower than the 24R method. Table 2 displays all means and differences. Pearson’s correlation analyses revealed significant, medium, positive associations between control and mfood log total energy (*p* = 0.03), carbohydrate (*p* = 0.02), and protein (*p* = 0.04) intakes. A significant, medium, positive association was also noted between the control and 24R total energy intake (*p* = 0.01); additionally, a strong, positive association between the control and 24R carbohydrate intake was observed (*p* < 0.001, Table 3). Significant, medium, positive associations were also observed between 24R and mfood log mean total energy (r = 0.59, *p* = 0.03) and protein (r = 0.62, *p* = 0.02) intakes.

### 3.3. Level of Agreement between Variables

**Figure 2:**
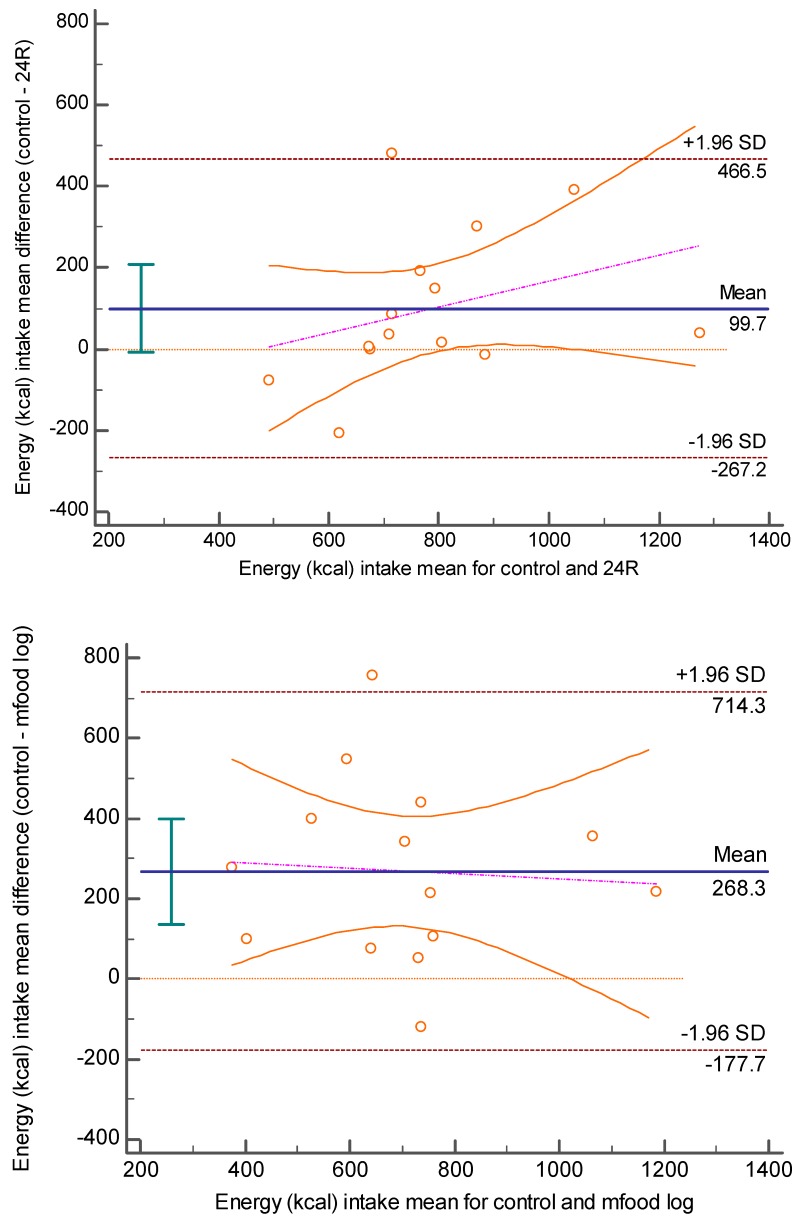
Bland Altman plot with regression lines for mfood log (control—mfood log) and 24R (control—24R).

Statistically, both the mfood log and 24R methods are considered to be in agreement with the control as the values are within the upper and lower confidence intervals of the mean +/− standard deviation, respectively (See Figure 2). The regression line of differences was insignificant for 24R (β = 0.32, *p* = 0.25) and mfood log (β = −0.07, *p* = 0.83). Practically, the limits are wide considering the mean differences were 177.7 to 267.2 kcals below the mean and 466.5 to 714.2 kcals above the mean for 24R and mfood log, respectively.

### 3.4. Survey Descriptives

While responses to the likelihood of using the mfood log on a daily basis had a wide range, with a lower mean than ease of use, the mfood log was generally reported as easy to use (see Table 4). Only three participants (of the 15) reported having used another dietary intake mobile application and all three reported having previously used MyFitnessPal. Pearson’s correlation analyses revealed a significant inverse association (r = −0.60, *p* = 0.03) between ease of use and mean energy intake differences between control and mfood log; no association (r = −0.45, *p* = 0.11) was identified between the mean difference and likelihood of use. 

## 4. Discussion

The purpose of this study was to examine the reporting accuracy of an mfood log and in comparison with a control meal and gold standard food recall method (24R). The study results showed that participants significantly underreported energy intake when data were entered into the mfood log compared to what was observed at the control meal. The 24R method also underreported, but was not significantly different from the control meal; energy intake was also not significantly different between the 24R and mfood log methods. Limits of agreement were similar between both control and mfood log and control and 24R, with no statistical importance, but may have practical limitations. 

While there is limited research examining the dietary intake reporting accuracy of mobile applications, a similar study has found strong correlations (r = 0.69–0.86, *p* < 0.001) between energy macronutrients captured via a food diary mobile application and 24 h recalls over two days [13]. Another study examined the accuracy of a mobile application to measure the intake of food groups and found strong correlations (mean r = 0.79, range: 0.69–0.88) between a 3-day, 24-h recall and the mobile application [14]. Similarly, our study did find a significant linear relationship between the control meal and both mfood log and 24R methods. Our results also align with another study that reported small mean differences and medium correlations between the 24R and mfood log [15]. 

Reported results showed promise for estimation of group means and thus, group dietary assessment with no proportional bias; however, further research is warranted for individual dietary assessment. Very few studies have examined the level of agreement using the Bland Altman plot; however, one study did examine Bland Altman plots and from a practical perspective, reported wide agreement between a mobile food log and 24R (their method of comparison) [15]. While our study indicated that statistically, the methods could be used interchangeably, from a practical standpoint, the caloric range would have definite implications for inaccuracy at the individual level. It is difficult to directly compare the two studies since our data represents one meal, whereas Carter et al. [15] examined dietary data for an entire day and thus, multiple meals. 

Our sample was largely female, two-thirds African American, and mostly overweight/obese. Mean values for both the mfood log and 24R reflected under-reporting of energy intake compared to the control method. In the United States, under-reporting of energy intake has been associated with female sex, non-Hispanic blacks, and overweight and obesity [16]. While it is difficult to determine these impacts on reporting in this study due to the relative homogeneity of the sample, this study provides key preliminary data toward the improvement of intake reporting using mfood logs among these populations. 

As for the acceptance of mobile applications for dietary assessment, our results indicated a general acceptance for ease and likelihood of using the mfood log. The findings correspond with research reporting participant preference for using a mobile application to log food intake instead of paper/pen methods [17]. A 2014 review of dietary assessment found that user satisfaction was high for six studies using mobile phones for dietary assessment, with one study reporting a low user satisfaction [10]. Two newer studies also corroborate with the general acceptance of mobile food records among adults, including in a community setting [18,19]. 

Sharp and Allman-Farinelli [10] found three predominant methods for dietary intake assessment, including a mobile phone electronic food diary (similar to our method), food photograph recall aids, analysis of food photographs by trained dietitians, and automated food photograph or video analysis. Compared to conventional methods (paper/pencil), reliability and validity were similar, but not inferior, to mobile methods; however, participant satisfaction and preference for mobile methods were higher [10]. While it seems that participant satisfaction with mobile dietary assessment tools echoes throughout the literature, this may not directly translate to implications for participant burden. Among adolescents reporting general satisfaction with a mobile dietary assessment tool, the majority (70%) of participants reported that the use of the application was burdensome or it was difficult to remember to record food intake [19]. Our own results suggest that those who found the application easier to use also had a smaller mean energy difference between the control method and mfood log. 

As it pertains to dietary self-monitoring, research has shown that the use of mobile applications versus conventional (typically paper records) techniques may result in better self-monitoring adherence and improvements in dietary intake and anthropometrics [20]. Clearly, more research is needed in larger, more diverse samples, and longer duration studies to determine the feasibility and validity of using mobile food diaries or logs to assess dietary intake at the group and individual levels. Additionally, mobile technologies offer other advantages, such as standardized and automated, real-time data entry [21]. Research should focus on innovative, mobile-supported solutions to reduce participant underreporting, improve food log adherence and ease of use, and mitigate portion size estimation error. There are several limitations of the reported study that must be noted. Sample size was small, with only 14 participants and a convenience sample; therefore, results may not be generalizable to a larger population, which is a typical limitation [10]. Due to the controlled component of the study, our data only captures data entry at one meal and not multiple meals or days. As a result, our methods also do not account for a possible learning effect that may have improved reporting for both the 24R and mfood log methods. Additionally, the controlled nature of the study lends itself to smaller sample sizes, which is a strength and limitation similar in the limited related research [15,22]. Lastly, similar to another study [15], we cannot determine how available nutrition information, that is viewable while using the mfood log, impacts dietary intake reporting. 

Our study is one of the few studies that examines Bland Altman plot and Pearson correlation analyses for the validity of dietary intake reporting using a mobile application dietary assessment food log tool. The results warrant further exploration to determine the ability of mobile technology to serve as a valid and reliable method for dietary data collection to be used for intervention and epidemiological purposes. Future studies should consider larger, more generalizable sample sizes, as well as the incorporation of gold standard assessment or controlled methods for comparison and longitudinal designs. 

## Figures and Tables

**Table 1:** Race, gender, and body mass index class of participants, *n* = 15.

Characteristic	n	%
Race		
Caucasian	3	21.4
African American	9	64.3
Other Race	2	14.1
Gender		
Female	11	78.6
Male	3	21.4
Body Mass Index Class		
Normal (18.0–24.9 kg/m^2^)	2	14.3
Overweight (25.0–29.9 kg/m^2^)	2	14.3
Obese Class 1 (30.0–34.9 kg/m^2^)	4	28.6
Obese Class 2 (35.0–39.9 kg/m^2^)	4	28.6
Obese Class 3 (>/=40.0 kg/m^2^)	2	14.3

**Table 2:** Mean energy and macronutrient intake of each method and difference from control meal, *n* = 14.

Reporting Method	Energy Intake (Kcal) Mean (±SD)	Energy Intake (Kcal) Mean Difference from Control	Carbohydrate Intake (g) Mean (±SD)	Carbohydrate Intake (g) Mean Difference from Control	Protein Intake (g) Mean (±SD)	Protein Intake (g) Mean Difference from Control	Fat Intake (g) Mean (±SD)	Fat Intake (g) Mean Difference from Control
Control Meal	838.88 (239.47)		108.54 (34.99)		36.16 (10.34)		28.57 (8.62)	
mFood Log	570.57 (252.22) ^1^	268.31 (227.53)	100.50 (46.60)	8.04 (38.02)	15.79 (7.87) ^1,2^	20.37 (8.91)	9.07 (7.94) ^1,2^	19.51 (10.76)
24R	739.21 (183.32)	99.67 (187.17)	104.84 (27.11)	3.70 (20.38)	29.23 (13.45) ^2^	6.93 (12.53)	22.15 (7.94) ^1,2^	6.43 (8.92)

^1^ Significant mean difference between mfood log or 24R and control methods (*p* < 0.05); ^2^ Significant mean difference between mfood log and 24R methods (*p* < 0.01); Abbreviations: g, grams; Kcal, kilocalories; SD, standard deviation.

**Table 3:** Pearson’s correlation analyses (with reported r- and *p*-values) of total energy and macronutrient intake between control, mfood log, and 24R methods.

	r-Value (*p*-Value)			
	Control Meal			
	Total Energy (Kilocalories)	Carbohydrate Intake (Grams)	Protein Intake (Grams)	Fat Intake (Grams)
mFood Log				
Total Energy (Kilocalories)	0.57 (0.03) ^1^			
Carbohydrate Intake (grams)		0.60 (0.02) ^1^		
Protein Intake (grams)			0.55 (0.04) ^1^	
Fat Intake (grams)				0.16 (0.59)
24R Method				
Total Energy (Kilocalories)	0.64 (0.01) ^1^			
Carbohydrate Intake (grams)		0.81 (<0.001) ^1^		
Protein Intake (grams)			0.47 (0.09)	
Fat Intake (grams)				0.42 (0.13)

^1^ Significant Pearson’s correlation (r-value) coefficients.

**Table 4:** Survey results for ease and likelihood of use for the Bridge2U mfood log, *n* = 15.

Survey Item	Response Range ^1^	Mean
Ease of use		
	3–10	7.5
Likelihood of Daily Use		
	1–9	5.9

^1^ Higher numerical value indicates a positive response (for example, “most likely”).

## References

[1] Thompson F.E., Kirkpatrick S.I., Subar A.F., Reedy J., Schap T.E., Wilson M.M., Krebs-Smith S.M. (2015). The national cancer institute’s dietary assessment primer: A resource for diet research. J. Acad. Nutr. Diet..

[2] Thompson F.E., Subar A.F., Coulston A.M., Boushey C.J., Ferruzzi M.G. (2017). Dietary assessment methodology. Nutrition in the Prevention and Treatment of Disease.

[3] Institute of Medicine (US) Committee on Military Nutrition Research (1997). Emerging Technologies for Nutrition Research.

[4] Moshfegh A.J., Rhodes D.G., Baer D.J., Murayi T., Clemens J.C., Rumpler W.V., Paul D.R., Sebastian R.S., Kuczynski K.J., Ingwersen L.A. (2008). The US department of agriculture automated multiple-pass method reduces bias in the collection of energy intakes. Am. J. Clin. Nutr..

[5] Subar A.F., Freedman L.S., Tooze J.A., Kirkpatrick S.I., Boushey C., Neuhouser M.L., Thompson F.E., Potischman N., Guenther P.M., Tarasuk V. (2015). Addressing current criticism regarding the value of self-report dietary data. J. Nutr..

[6] Samuel-Hodge C.D., Fernandez L.M., Henríquez-Roldán C.F., Johnston L.F., Keyserling T.C. (2004). A comparison of self-reported energy intake with total energy expenditure estimated by accelerometer and basal metabolic rate in African-American women with type 2 diabetes. Diabetes Care.

[7] Voss S., Kroke A., Klipstein-Grobusch K., Boeing H. (1997). Obesity as a major determinant of underreporting in a self-administered food frequency questionnaire: Results from the EPIC-Potsdam Study. Z. Ernahrungswiss.

[8] Bedard D., Shatenstein B., Nadon S. (2004). Underreporting of energy intake from a self-administered food-frequency questionnaire completed by adults in Montreal. Public Health Nutr..

[9] Stumbo P.J. (2013). New technology in dietary assessment: A review of digital methods in improving food record accuracy. Proc. Nutr. Soc..

[10] Sharp D.B., Allman-Farinelli M. (2014). Feasibility and validity of mobile phones to assess dietary intake. Nutrition.

[11] National Agricultural Library USDA Food Composition Databases. https://ndb.nal.usda.gov/ndb/search/list?home=true.

[12] 24-hour Dietary Recall (24HR) At a Glance. https://dietassessmentprimer.cancer.gov/profiles/recall/.

[13] Carter M.C., Burley V.J., Cade J.E. (2013). Development of ‘My Meal Mate’—A smartphone intervention for weight loss. Nutr. Bull..

[14] Rangan A.M., Tieleman L., Louie J.C.Y., Tang L.M., Hebden L., Roy R., Kay J., Allman-Farinelli M. (2016). Electronic Dietary Intake Assessment (e-DIA): Relative validity of a mobile phone application to measure intake of food groups. Br. J. Nutr..

[15] Carter M.C., Burley V.J., Nykjaer C., Cade J.E. (2013). ‘My Meal Mate’ (MMM): Validation of the diet measures captured on a smartphone application to facilitate weight loss. Br. J. Nutr..

[16] Murakami K., Livingstone M.B.E. (2015). Prevalence and characteristics of misreporting of energy intake in US adults: NHANES 2003–2012. Br. J. Nutr..

[17] Carter M.C., Burley V.J., Nykjaer C., Cade J.E. (2013). Adherence to a smartphone application for weight loss compared to website and paper diary: Pilot randomized controlled trial. J. Med. Internet Res..

[18] Fowler L.A., Yingling L.R., Brooks A.T., Wallen G.R., Peters-Lawrence M., McClurkin M., Wiley K.L., Mitchell V.M., Johnson T.D., Curry K.E. (2018). Digital food records in community-based interventions: Mixed-Methods pilot study. JMIR mHealth uHealth.

[19] Lee J.-E., Song S., Ahn J., Kim Y., Lee J. (2017). Use of a mobile application for self-monitoring dietary intake: Feasibility test and an intervention study. Nutrients.

[20] Lieffers J.R.L., Hanning R.M. (2012). Dietary Assessment and Self-monitoring: With Nutrition Applications for Mobile Devices. Can. J. Diet. Pract. Res..

[21] Forster H., Walsh M.C., Gibney M.J., Brennan L., Gibney E.R. (2016). Personalised nutrition: The role of new dietary assessment methods. Proc. Nutr. Soc..

[22] Arab L., Estrin D., Kim D.H., Burke J., Goldman J. (2011). Feasibility testing of an automated image-capture method to aid dietary recall. Eur. J. Clin. Nutr..

